# Performance evaluation and quantitative comparison of two 4DCT imaging respiratory systems using deformable image registration

**DOI:** 10.1002/acm2.70279

**Published:** 2025-10-10

**Authors:** Ali Al‐Zein, Rawan H. Naim, Wassim Jalbout, Bilal H. Shahine

**Affiliations:** ^1^ Radiation Oncology Department American University of Beirut Medical Center Beirut Lebanon

**Keywords:** 4DCT image reconstruction, deformable image registration, GateCT, pressure sensor ANZAI

## Abstract

**Purpose:**

Improved accuracy in 4DCT imaging and precise targeting of tumors contribute to more effective and targeted radiation therapy. This study focuses on evaluating the accuracy of utilizing the GateCT (VisionRT Ltd, London, United Kingdom) in comparison with a pressure sensor system (ANZAI Medical Co., Ltd., Shinagawa, Tokyo) to provide 4DCT with respiratory information.

**Methods:**

A dynamic breathing phantom enclosing three spheres (A, P, and R) of different densities was enrolled to produce breathing patterns tracked by the two systems. Image sets for three breathing phases obtained based on GateCT and ANZAI systems were analyzed using deformable registration by deforming the three‐phase image sets with the static image sets. Our deformable registration approach revealed how far different phase image sets were from the quantified by various metrics, such as dice similarity coefficient (DSC), mean surface distance (MSD), absolute volume estimation, mean Jacobian, and Warp.

**Results:**

Results indicated DSC values greater than 0.90 across all phases and spheres for both respiratory systems, with mean DSC values for spheres A, P, and R of 0.980 versus 0.977, 0.977 versus 0.976, and 0.977 versus 0.976 for GateCT and ANZAI systems, respectively. MSDs for both systems were consistently less than 2 mm across all spheres and phases. Furthermore, the mean volume estimation error for both systems, relative to the static, exhibited statistical insignificance (*p* > 0.05). Friedman test revealed significant differences in median Jacobian, and median Warping between the two systems (*p* < 0.05).

**Conclusions:**

In addition to the effectiveness of deformable image registration in the quantification of respiratory system performance, both systems exhibited comparable performance in providing 4DCT with respiratory information.

## 1 INTRODUCTION

Breathing‐induced motion poses a significant challenge in the context of medical imaging, particularly during three‐dimensional computed tomography (3DCT) of the thorax and abdomen in free‐breathing conditions. Intrafraction motion provoked by the movement of skeletal‐muscular, respiratory, gastrointestinal, and cardiac systems, is a significant issue to be managed during 3DCT and treatment planning as the target volume and organs at risk (OARs) are subjected to geometry variations.^1^ The treatment planning and the dose delivered during radiotherapy are influenced by these variations in terms of motion distortion and image artifacts.^2^ Therefore, an unintended radiation dose distribution may be administered to the target volume and nearby organs at risk.^3^, ^4^ Clinical strategies were employed to address the challenges of tumor motion during treatment planning and radiation delivery. The strategies include: (1) expanding the margin of the target volume by considering its internal motion to generate the internal target volume (ITV),^5^ (2) applying abdominal compression to limit tumor movement,^1^ (3) utilizing breath‐hold techniques,^1^, ^6^ (4) implementing tumor tracking,^7^, ^8^ and (5) employing breathing‐adapted gating methods to synchronize radiation delivery with specific phases of patient breathing.^1^, ^9^, ^10^ The integration of respiratory monitoring devices and imaging techniques such as four‐dimensional computed tomography(4DCT) is crucial in the treatment planning of patients undergoing thoracic and abdominal radiotherapy as these modalities help in defining adequate field margins around the tumor volume and prevent unnecessary radiation exposure to OARs by accounting for variations in the position of organs and tumors during different phases of the breathing cycle.^11^ Radiation oncology departments worldwide reported the use of various respiratory monitoring devices, the most popular sensor used for 4DCT is the respiratory gating system AZ‐733 V (ANZAI Medical Co., Ltd., Shinagawa, Tokyo) composed of a pressure sensor and a belt surrounding the patient's moving structure.^12^, ^13^ However, the use of ANZAI has its drawbacks in terms of its reliance on a single point for recording the surface breathing pattern location and tightness level, resulting in inconsistent results and potentially leading to distortion in the 4DCT reconstruction.^14^ Many studies compared the reliability and accuracy of various respiratory gating systems, but none employed deformable image registration as a quantitative comparison method. A study was conducted by Kauweloa et al.^2^ to compare the surface‐guided GateCT system (VisionRT Ltd, London, United Kingdom) versus the Real‐Time Position Management (RPM) system (Varian, Palo Alto, CA), by evaluating phase and amplitude accuracy. They found that GateCT system demonstrated consistency in temporal and phase tracking but exhibited limitations in accurately tracking absolute abdominal positions. Another perspective was introduced by Heinz et al.^15^ in combining ANZAI Respiratory Gating System and C‐RAD Sentinel (C‐RAD AB, Uppsala, Sweden) with an Aquilion Large Bore CT scanner (Toshiba Medical System Group, Tokyo, Japan). Various parameters were used to analyze both systems such as trigger compatibility, signal‐to‐noise ratio (SNR), signal linearity, signal amplitude, noise, and time resolution on clinical applications. Their investigation found that 4DCT reconstruction outcomes were improved when using a Toshiba CT scanner in conjunction with the ANZAI system versus C‐RAD Sentinel. This is because the ANZAI system was compatible with image sorting and trigger release procedures of the mentioned CT scanner. Considering these findings, there was a strong recommendation for integrating a cycle‐based trigger option into the c4D software for Toshiba CT scanners, alongside recommending visual feedback of the respiratory signal. This enhancement ensures amplitude stability, enables comparable triggers to cycle‐based methods, and addresses irregular breathing issues in 4DCT reconstructions. Another comparative performance assessment of SimRT (VisionRT Ltd., London, United Kingdom) versus ANZAI (Anzai Medical Co., Ltd., Shinagawa, Tokyo) was performed by Qubala et al.^14^ Many parameters were evaluated and compared to the ground truth, such as Pearson correlation (PC), mean absolute deviation (MAD), breathing amplitude, and period. In contrast to Anzai, SimRT demonstrated superior breathing tracking capabilities, remaining accurate and stable regardless of variations in breathing pattern, amplitude, or period. This led to consistently precise temporal and spatial accuracy with SimRT.

Moreover, they found that SimRT demonstrated superior accuracy in reproducing breathing signals, with a lower Mean Absolute Deviation (MAD) range compared to ANZAI, for regular and irregular breathing patterns.

At our center, the respiratory gating system ANZAI and GateCT respiratory monitoring systems were utilized to track the breathing pattern for 4DCT reconstruction. The purpose of this research is to evaluate the efficacy of the GateCT against ANZAI system in the context of 4DCT reconstruction. To compare the two systems, deformable image registration (DIR) methods were used in the assessment. DIR can evaluate local 3D respiratory motion anatomy especially for voxel‐by‐voxel mapping of medical images that have large scale deformations, thus enhancing simulation, planning, and execution of accurate treatment delivery. Image registration is used widely in radiotherapy, including segmentation, adaptive treatment planning, image guided radiotherapy and response assessment. It is crucial to correlate identical anatomical points in both data sets being fused. The alignment of anatomy or features delineation are crucial in determining the accuracy of image registration^16^ 4DCT reconstructions with different breathing amplitudes were evaluated using various methods to quantitatively assess differences between the two systems such as dice similarity coefficient (DSC), mean surface distance (MSD), absolute volume change, Jacobian determinant, and Warp. Our work was based on the recommendation of the American Association of Physicists in Medicine Task Group 132 (AAPM TG132)^17^ that highlights the use of image registration, fusion algorithms, and techniques in radiotherapy. The couch speed as a function of the weight was also investigated to ensure the proper tracking of GateCT system at different couch load.

## 2 MATERIALS AND METHODS

### 2.1 Siemens CT scanner

All measurements were performed on the SOMATOM Confidence helical multi‐slice CT scanner (Siemens Healthineers GmbH, Erlangen, Germany). In 4DCT studies, the sorting of CT images was done through a phase‐based algorithm. The algorithm used enabled image reconstruction during specific breathing phases, in alignment with the recorded breathing patterns provided by the respiratory monitoring systems. The respiratory monitoring system sends a trigger to the CT scanner in each breathing cycle. The CT scanner relies on these trigger pulses to synchronize image acquisition with the specific phases of the respiratory cycle. The acquired images are then sorted into different phases of the breathing cycle.^15^ Thus, the imaging process acquires distinct points called bins with each breathing cycle. In our study, six groups of 4DCT datasets were created: three for ANZAI and three for GateCT system, all having 1.5 mm slice thickness.

### 2.2 Respiratory Monitoring Systems

**FIGURE 1:**
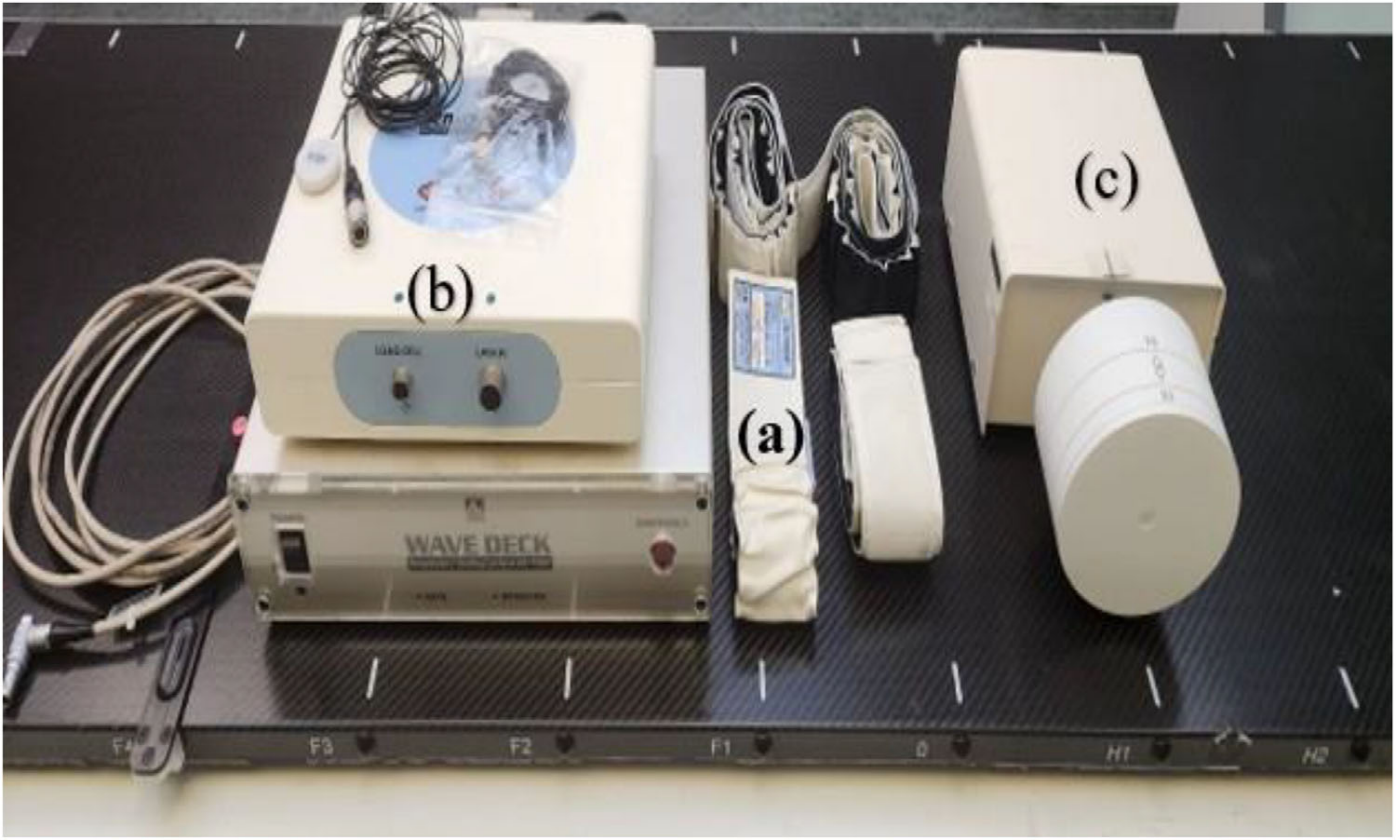
Components of the ANZAI system: (a) different sizes of the fixation belts, (b) Anzai AZ‐733V

**FIGURE 2:**
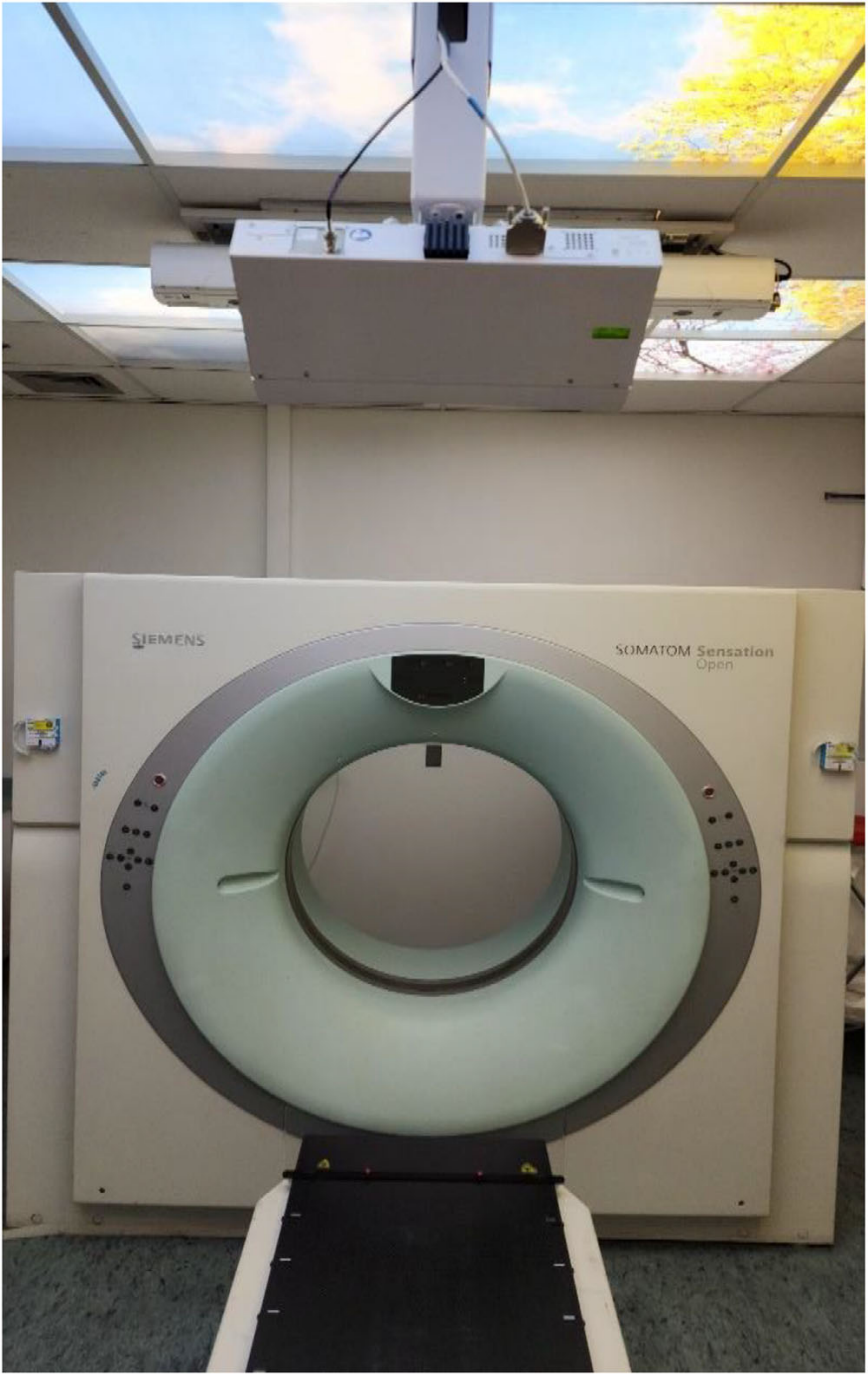
The GateCT system with centrally positioned 3D camera unit mounted at the ceiling.

We compared two monitoring systems: The AZ‐733 V system and the GateCT system. The AZ‐733 system illustrated in Figure 1 is composed of a pressure sensor (30 mm diameter and 9.5 mm thickness) attached to a fixation belt that is fastened to the body site such as abdomen. The breathing pattern can then be determined and sent to the CT console in real time by estimating the pressure induced by the fluctuation in tidal body volume.^14^ It operates with a fixed sampling rate of 40 Hz. The second system, GateCT, utilizes a centrally positioned 3D camera unit mounted in the ceiling that is focused on the central scanning plane of the CT scanner (Figure 2). It tracks the respiratory signal from the movement of the 3D surface of the patient during 4DCT data acquisition. The system was synchronized with the CT scanner for 4DCT reconstruction.

### 2.3 Breathing Phantom

The breathing phantom (AZ‐733 V) was used to simulate the breathing pattern of the lungs. It is composed of three spheres (anterior sphere A—made of rubber: 4.7 cm diameter, ‐161.6 HU; posterior sphere P—made of acrylic: 5 cm diameter,118.2 HU and a Right sphere R—made of wood: 5 cm diameter, ‐359 HU).

### 2.4 GateCT system and 4DCT Synchronization

The stimulated‐breathing pattern for the phantom was tracked by the GateCT system. A scout image of the phantom with a defined region of interest was acquired, and the respiratory signal was visualized on the GateCT system. It is important to note that the movement of the couch during the procedure poses a crucial challenge regarding the accuracy of the recorded respiratory signal. Dealing with the latter challenge requires synchronization between the recorded GateCT signal and the couch movement. This issue is dealt with in Sections 2.4 and 3.6. When GateCT displays the breathing signal, it determines the region where the X‐ray was active and synchronized with the movements of the couch.

### 2.5 Couch speed versus weight

Our objective was to investigate the relationship between patient weight and couch speed. It was hypothesized that the couch speed is independent of the patient's weight. To test the hypothesis, a fixed couch speed value of 3.0 mm/s and a load of 0, 52, and 103 kg were applied to the CT couch for proper evaluation. This means that for a given speed, and different loads, the breathing signal of the phantom must be tracked appropriately. The hypothesis was deemed null hypothesis if the breathing signal exhibited amplitude variation and true if otherwise.

### 2.6 Data acquisition

This research examined the response of the two respiratory systems using the AZ‐733 V breathing phantom. Both systems tracked the breathing phantom and generated 3DCT datasets at different phases (0%, 50%, and 100% inhale). A CT scan for the static phantom was also acquired.

### 2.7 Deformable Image Registration (DIR) software

VELOCITY 3.2.0 (Varian Medical Systems, Palo Alto, CA) allows display, annotation, volume rendering, rigid registration, and deformable fusion of medical images of different modalities. A predefined HU threshold was used to delineate the three spheres (A, P, and R) in the 4DCT data sets of the ANZAI and GateCT systems at the three phases defined above. The Hounsfield Unit (HU) threshold utilized to auto‐delineate the three spheres on the static CT was adjusted to ensure that the delineated volume matches the real volume for each sphere. Accordingly, the Hounsfield Unit (HU) thresholds were established at ‐710 HU for sphere A,

‐775 HU for sphere P, and ‐614 HU for sphere R. DIR was performed between each deformed sphere at each breathing phase of GateCT and ANZAI systems.

### 2.8 DIR Systematic and Random Errors Analysis

AAPM TG132 recommends metrics such as target registration error (TRE) for estimating DIR accuracy. TRE assesses the internal alignment of images, and it is calculated as the error of the target fiducial markers position following registration. In this study, the center of the inferior last slice in each sphere was considered as our fiducial marker. This location was chosen in particular as it constitutes the maximum positional error that can result from DIR operation when compared to more stable location such as the center of the sphere. Deformed sphere and still sphere were overlaid, and TRE of fiducial pairs was calculated for both GateCT and ANZAI systems.

### 2.9 Dosimetric indication of DIR geometric accuracy

The 3DCT datasets acquired at different phases (0%, 50%, and 100% inhale) were utilized to construct and internal target volume (ITV) for all 3 spheres. Highly conformal treatment plans to cover ITVs were generated on Eclipse treatment planning system (TPS) (Varian Medical Systems, Palo Alto, CA). TRE shift results were applied on the isocenter location latter plans to study the dosimetric impact of DIR systematic errors. To eliminate effects of dose variations due to inhomogeneities in the phantom, no heterogeneity correction was applied to our plans. Parameters such as the isodose covering 95 percent of the ITV volume (D95), the percentage volume covered by 100 percent of prescription dose (V100), and ITV mean dose (Dmean) were compared because of the isocenter systematic shift. These tests were repeated for scans obtained for both GateCT and ANZAI systems.

### 2.10 Assessment Metrics and Statistical Analysis

Deformable image registration was followed by the evaluation of the two respiratory tracking systems using a set of QA metrics. The change in shape and volume of the contours were compared for different phases against the data obtained from the CT dataset of the phantom in static position. Thus, the metrics, which are used for the purpose of ensuring the quality of deformable registration, were employed to compare GateCT and ANZAI systems performance. Anatomical agreement and deformation field metrics were the two sets of metrics used in the research as illustrated in Table 1. Anatomical agreement metrics evaluate how well the aligned objects resulting from different phases match their corresponding static scan. Metrics such as dice similarity coefficient (DSC), mean surface distance (MSD), and absolute volume estimation (AVE) were used to assess the degree of overlap or agreement for each sphere at different phases. The deformation field represents the spatial transformation applied to the secondary CT to align with the primary one. It essentially describes how each voxel in the moving objects is mapped to its corresponding location in the static CT of the still phantom. The quality of the deformation field was evaluated based on metrics such as Jacobian Determinant and Warping parameter. In this study, statistical comparisons were performed employing both parametric and nonparametric tests to compare metrics derived from the GateCT and ANZAI systems. Given the nature of the deformation field data and potential violations of normality assumptions, the Friedman test (2‐way ANOVA) was utilized to assess differences between the two systems. The anatomical metrics were analyzed using parametric *t*‐test. The statistical analysis was carried out using IBM SPSS Statistics, Version 29.0 (IBM Corp., Armonk, NY). A *p*‐value of less than 0.05 indicates that the two systems are statistically significant.

**TABLE 1:** Systems evaluation scheme using DIR metrics to quantitatively assess the performance of ANZAI and GateCT.

Systems Evaluation Scheme using DIR Metrics	
**Anatomical Agreement**	**Deformation Field**
Dice Similarity Coefficient Mean Surface Distance Absolute Volume Estimation	Jacobian Determinant Warp

## 3 RESULTS

### 3.1 DSC

**FIGURE 3:**
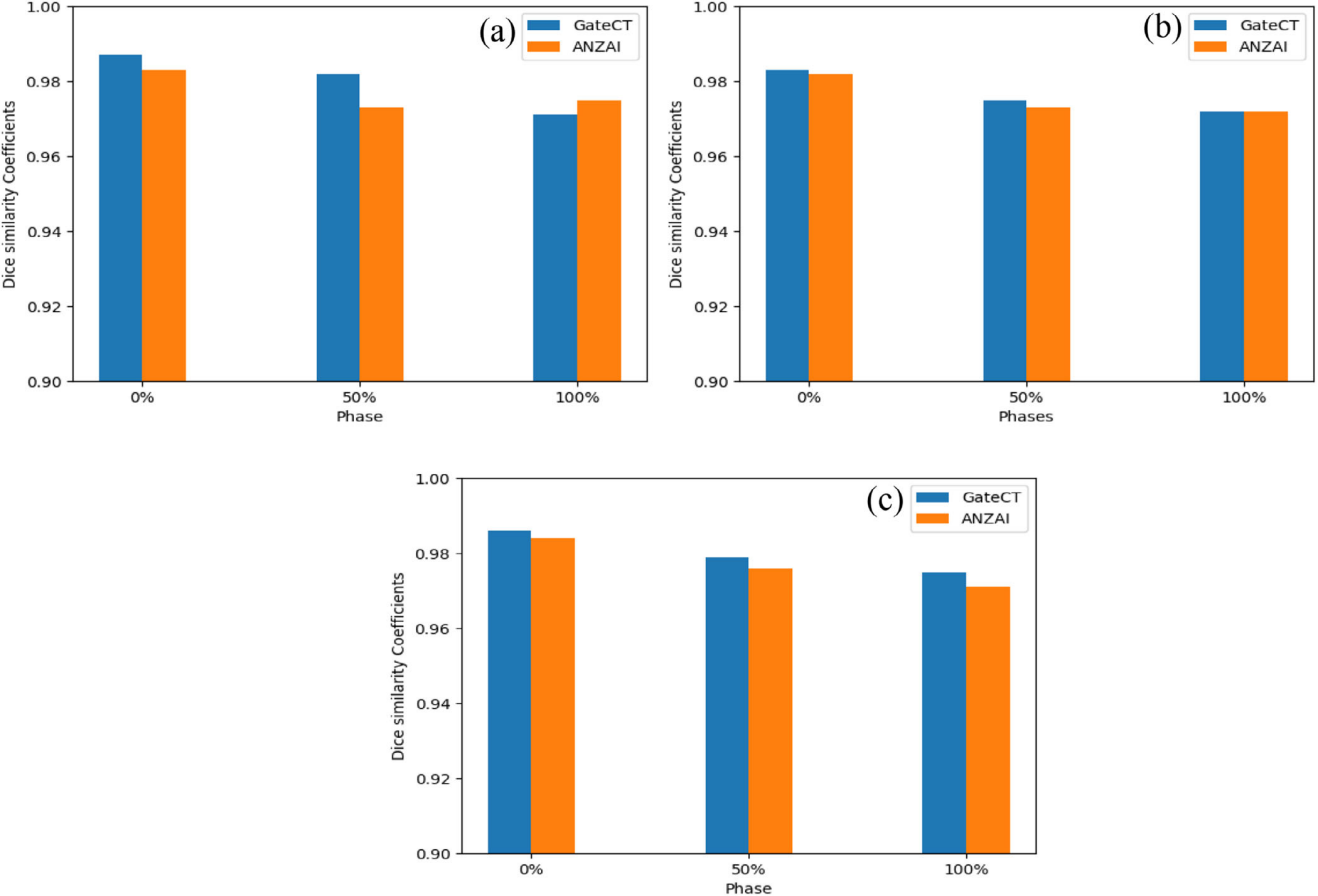
Dice similarity Coefficients for sphere (a) A, (b) P, and (c) R, utilizing GateCT versus ANZAI system.

DSC illustrated in Figure 3 showed how close the contours of the three spheres (A, P, and R) on the 4DCT scans, acquired using the ANZAI or GateCT systems, were to the static CT scan after deformable registration. It is noticeable that both systems had DSC close to 1 and were comparable in values across the three spheres and phases. The average DSC across the different phases for spheres A, P, and R exhibited values of 0.980 vs. 0.977, 0.977 vs. 0.976, and 0.977 vs. 0.976 for GateCT and ANZAI systems, respectively. The small variation between the mean DSCs for different spheres suggests that the two systems are almost identical, and the DSC metric difference between the two systems was statistically insignificant with *p* > 0.05.

### 3.2 MSD

Results from the analysis revealed that both systems yielded relatively comparable MSDs for the three spheres across the 4DCT phases, as shown in Table 2. Moreover, comparable standard deviations imply that both systems exhibit similar levels of variability or spread in their estimates. This consistency suggests that neither system was more prone to produce outliers compared to the other. Statistically, across all phases and spheres, there were no significant differences in the MSDs between the two systems with a *p*‐value > 0.05.

**TABLE 2:** MSD for sphere A, P, and R at 0%, 50%, and 100% inhalation phases for GateCT versus ANZAI system.

		MSD (mm) ± σ		
Sphere	System	0 % In	50 % In	100 % In
**A**	**GateCT**	0.2 ± 0.3	0.2 ± 0.4	0.3 ± 0.5
	**ANZAI**	0.2 ± 0.3	0.3 ± 0.5	0.3 ± 0.5
**P**	**GateCT**	0.3 ± 0.5	0.3 ± 0.6	0.4 ± 0.6
	**ANZAI**	0.3 ± 0.6	0.4 ± 0.7	0.4 ± 0.7
**R**	**GateCT**	0.2 ± 0.4	0.3 ± 0.5	0.3 ± 0.4
	**ANZAI**	0.3 ± 0.5	0.3 ± 0.6	0.4 ± 0.5

### 3.3 AVE

AVE for the three spheres at each breathing phase were compared to their static CT scan volumes, as shown in Table 3. All their volume estimates were done using auto‐delineation with threshold values as specified in Section 2.7. The greatest percent volume deviation from the static CT volumes occurred with ANZAI (+ 8.8%) for sphere R at 100% inhalation. In comparison, the corresponding percent volume discrepancy for the GateCT system for the same sphere and phase indicated a +7.3% deviation. The mean percentage volume discrepancy for the GateCT versus ANZAI systems with respect to the static CT values was 2.7 versus 2.5, 2.5 versus 1.3, and 4.6 versus 6.0 for spheres A, P, and R, respectively. However, these results were not significantly different, as demonstrated by the *t*‐test with *p* > 0.05.

**TABLE 3:** GateCT versus ANZAI volume discrepancies for sphere A, P, and R at 0%, 50%, and 100% inhalation phases as compared to the static CT volumes of 54.3 cm^3^, 65.5 cm^3^, and 65.5 cm^3^, respectively.

Sphere	Phase	GateCT	ANZAI
**A**	**0%**	53.7 (−1.1)	54.4 (+0.2)
	**50%**	55.3 (+1.8)	56.7 (+4.4)
	**100%**	57.1 (+5.2)	55.9 (+3.0)
**P**	**0%**	63.7 (−2.7)	65.2 (−0.4)
	**50%**	66.8 (+2.0)	66.2 (+1.1)
	**100%**	67.3 (+2.7)	67.1 (+2.4)
**R**	**0%**	66.5 (+1.5)	67.2 (+2.6)
	**50%**	68.7 (+4.9)	69.9 (+6.7)
	**100%**	70.3 (+7.3)	71.3 (+8.8)

### 3.4 Jacobian metrics (J)

After the deformable registration was done between the static CT and the 4DCT scans of the breathing phantom acquired by GateCT or ANZAI respiratory monitoring systems, the median Jacobian determinants were illustrated in Table 4. The median Jacobian values for the three spheres were around 1 at the three inhalation phases (0%, 50%, and 100%) for both systems. It is worth noting that the Jacobian distribution was not Gaussian distribution as determined by Kolmogorov Smirnov test and hence median values were chosen to compare the two systems.

**TABLE 4:** Median Jacobian and Jacobian range for ANZAI versus GateCT system. Bold figures indicate a winning respiratory system.

		Median Jacobian(Min‐Max)		
Sphere	System	0 % In	50 % In	100 % In
**A**	**GateCT**	1.07 (0.65–1.49)	**1.02**(0.49–1.56)	1.10 (0.57–1.69)
	**ANZAI**	**1.05** (0.57–1.53)	1.10(0.39–1.81)	**0.99** (0.53–1.46)
**P**	**GateCT**	1.03 (0.65–1.41)	**1.00**(0.39–1.62)	1.03 (0.41–1.65)
	**ANZAI**	**0.97** (0.63–1.31)	0.94(0.32–1.57)	**0.99** (0.33–1.66)
**R**	**GateCT**	1.20 (0.69–1.70)	**1.02**(0.42–1.62)	0.90 (0.16–1.59)
	**ANZAI**	**0.97** (0.52–1.43)	1.11 (0.40–1.82)	**0.90** (0.37–1.44)

### 3.5 Deformation Grid and Warping Parameter

**FIGURE 4:**
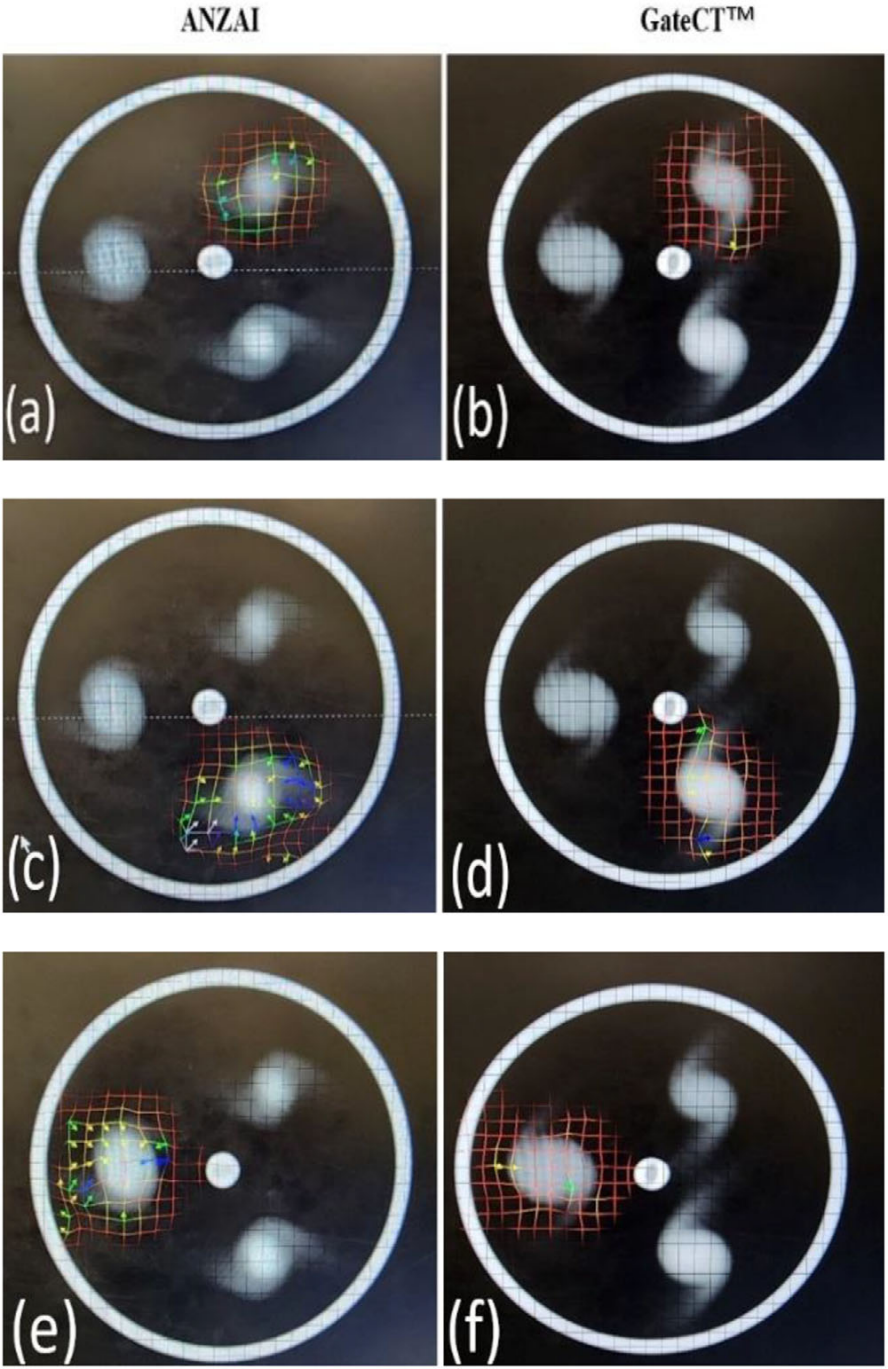
Deformation grid of the three spheres with respect to the static CT at 100% inhale **phase** for ANZAI versus GateCT systems**. Deformation vector fields are depicted for: (a) and (b) sphere A,** (c) and (d) sphere P, and (e) and (f) sphere R deformation.

A deformation grid showed how each voxel in the three spheres (A, P, and R) was deformed or warped to match the corresponding voxel in the static CT as shown in Figure 4. The grid consists of a set of vectors overlaid onto the static CT scan. Each vector indicated the direction and magnitude of the deformation applied to a specific voxel in the sphere for each phase of the 4DCT (by GateCT or ANZAI) to match static CT. Figure 4a showed that sphere A underwent significant deformations during the registration process at the 100% inhalation phase. A dense concentration of arrows suggested that voxels in sphere A in the 4DCT image by ANZAI had undergone substantial deformation to match its static CT. Nevertheless, when using the GateCT system, the same sphere experienced less deformation or discrepancy with respect to the static, as evidenced by the presence of a smaller number of arrows. Quantitatively and in terms of median Warping, the results were tabulated in Table 5. Statistical analysis revealed that both systems demonstrated significant difference in median Warping across each sphere, as evidenced by a *p*‐value less than 0.05.

**TABLE 5:** Median Warp for sphere A, P, and R at 0%, 50%, and 100% phases for GateCT versus ANZAI systems. Bold figures indicate a winning respiratory system.

		Median Warp (mm) (Min‐Max)		
Sphere	System	0 % In	50 % In	100 % In
**A**	**GateCT**	**0.8** (0–1.64)	1.1 (0–2.23)	**1.0** (0–2.01)
	**ANZAI**	0.9 (0–1.75)	**1.1** (0–2.15)	1.1 (0–2.21)
**P**	**GateCT**	0.8 (0–1.54)	**1.4** (0–2.85)	**1.0** (0–1.97)
	**ANZAI**	0.8 (0–1.54)	1.7 (0–3.42)	1.3 (0–2.61)
**R**	**GateCT**	0.7 (0–1.40)	1.4 (0–2.83)	1.3 (0–2.66)
	**ANZAI**	**0.7** (0–1.38)	**1.2** (0–2.44)	**1.1** (0–2.27)

### 3.6 Geometric Indication of Fiducial Motion on DIR TRE

Motion‐induced fiducial motion as depicted by the inferior slice of each sphere and its corresponding TRE was comparable for all 3 spheres and was in the order of 1.0 mm. This value of TRE was consistent for all 3 phases of motion and its standard deviation was found to be 0.6 mm. Both respiratory motion management systems were consistent in resulting target registration error of 1.0 ± 0.6 mm.

### 3.7 Dosimetric Indication of DIR Geometric Accuracy

Planned doses were not significantly affected by the above registration error. The three dosimetric parameters studied (D95, V100, and Dmean) derived from dose volume histogram analysis deviated within 1 percent between plans when isocenter was shifted 1.0 mm. This was repeated for both systems which showed matching results.

### 3.8 CT Couch Speed as a Function of Weight

**FIGURE 5:**
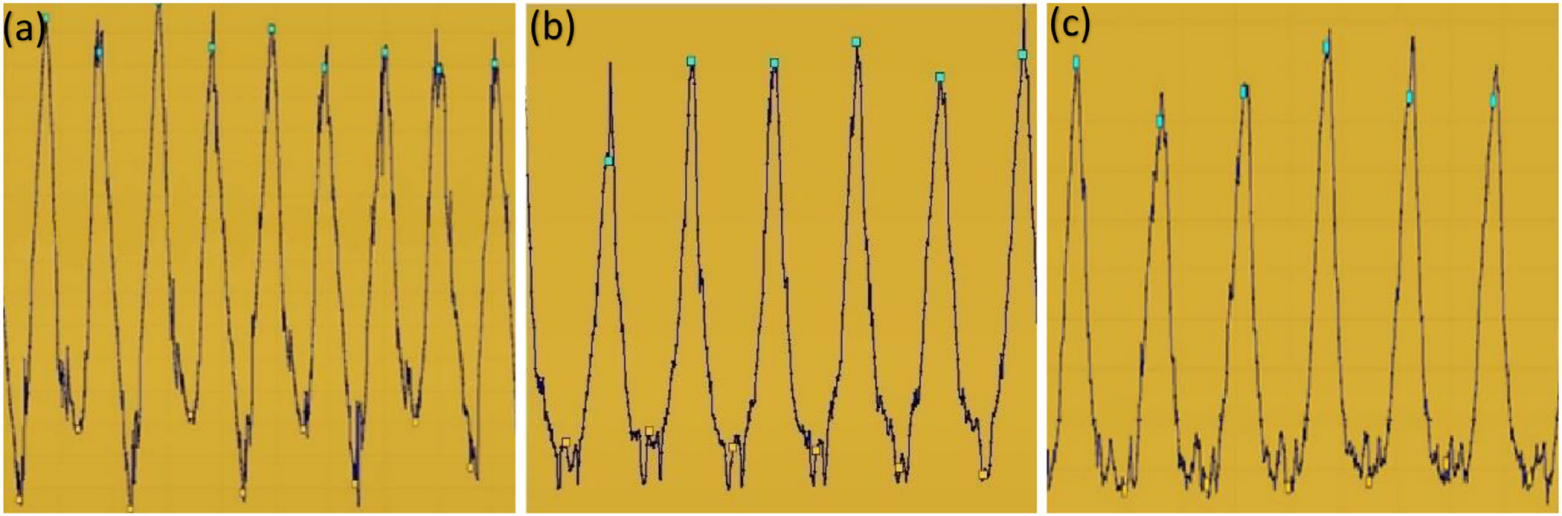
The breathing signal tracked by GateCT for 3.0 mm/s couch speed as a function of time for: (a) 0 kg, (b) 52 kg, and (c) 103 kg load on the CT couch.

Figure 5 shows the breathing pattern recorded by GateCT at a fixed couch speed of 3.0 mm/s with couch load of 0 (Figure 5a), 52 kg (Figure 5b), and 103 kg (Figure 5c). The results showed that the breathing signal was clearly detected, and its magnitude was consistent for all three loads.

## 4 DISCUSSION

Previous studies examined and compared the reproducibility, spatial, and temporal accuracy of breathing patterns and 4DCT reconstructions were examined to evaluate the volume discrepancy, shape deformation and tumor trajectory using different external respiratory monitoring systems.^2^, ^12^, ^14^, ^15^ In contrast to their methodology, our study employed deformable image registration as a means of comparing the two respiratory systems; that is, different phases of 4DCT scan of solid moving spheres versus static CT scan. This was conducted by evaluating various parameters such as dice similarity coefficients, mean surface distance, absolute volume estimation, Jacobian determinant, and Warping values.

### 4.1 Anatomical Agreement Metrics

DSC demonstrated that, for both systems, the three spheres (A, P, and R) contours overlapped with their static contours by more than 90% across all phases. Thus, the GateCT system showed comparable results to the ANZAI system, and both had dice similarity coefficients that were close to 1 in all three spheres and at all phases. This demonstrated that the static CT scan of the phantom and the 4DCT scans acquired using GateCT or ANZAI system of the three spheres overlapped within 90%. AAPM TG132 recommended a DSC tolerance typically in the range of 0.8 to 0.9. Setting a tolerance in this range ensures that the delineation result achieves a high level of overlap with the static contours.^17^ Overall, achieving DSC values consistently above 0.9 showed that both systems were comparable and within tolerance.

Moreover, our results revealed that the MSD was consistently less than 2 mm across the three spheres for both systems at all phases. This indicates that, on average, the contours surface for each sphere were within < 2 mm from the static contours for both systems. AAPM TG132 suggested a tolerance typically in the range of 2 to 3 mm. This means that for a delineation result to be considered acceptable, the MSD between the corresponding voxels on the two contour surfaces should be within this range. Both systems were accurately detecting the shape and boundaries of the spheres. Consistently achieving MSD values below 2 mm for both systems indicate consistency in the delineation results. This consistency is important for reproducibility and reliability in clinical settings. Additionally, the differences in MSDs (Table 2) between the ANZAI and GateCT were statistically insignificant with *p* > 0.05. This means that any observed difference was due to random variation rather than a true difference in performance between the systems. Based on the two metrics discussed above, while both ANZAI and GateCT systems had similar performance in terms of overall volume overlap (as indicated by the comparable DSCs), both systems tend to provide spatially accurate delineations of the boundaries and shapes of the three spheres, as evidenced by the tolerated MSD values.

Both ANZAI and GateCT systems exhibited discrepancies in estimating the spheres volumes in the moving phantom compared to the static phantom (Table 3). This suggests possible challenges in tracking respiratory motion when using both systems, especially during specific phases of the respiratory cycle. ANZAI tended to show smaller errors for certain spheres, while GateCT had its strengths at other spheres. The ANZAI system consistently exhibited greater overestimation of sphere R compared to the GateCT system across all three inhalation phases, with a mean discrepancy of 6.7% as opposed to 4.9% for GateCT at 50% inhalation for instance. The notable discrepancy observed in the estimation of the volume of sphere R can be attributed to its density, being composed of wood and accurate automatic delineation can be more challenging. Overall, the results suggested that while there was a slight discrepancy in the estimated volumes compared to their static volumes, the differences were statistically insignificant between the ANZAI and GateCT systems for absolute volume estimation for each sphere. This suggests that, on average, both systems performed comparably in absolute volume estimation across different breathing phases.

### 4.2 Deformation Field Metrics

Both systems revealed a median Jacobian around 1 (Table 4); thus, there was no significant expansion or contraction of the sphere voxels compared to the static sphere voxels. This shows the ability of both systems to accurately depict volumes during respiration, highlighting their potential for reliable and clinically relevant applications in 4DCT reconstruction. According to statistical analysis, the findings suggested that the two respiratory monitoring systems yielded different results in terms of local volume deformation. Specifically, a significant difference was observed (*p* < 0.05) across the three spheres, except for sphere R at 100% inhalation phase, where no statistically significant disparity was noted between the two systems. As depicted in Table 4, ANZAI demonstrated median Jacobian values closer to 1 in 6 cases, suggesting a tendency towards less local expansion or contraction compared of voxels to GateCT. Moreover, GateCT exhibited median Jacobian values closer to 1 in 3 cases at 50% inhalation phase, implying that this system led to less pronounced voxels deformation than ANZAI at this phase.

Furthermore, Negative Jacobian values can sometimes arise from registration errors, such as misalignments or inaccuracies in the deformation field.^17^ In our results, the deformable registration process did not produce any negative Jacobian values for both systems dataset, this suggests that the deformation applied by the registration process preserved local volumes and did not contract the voxels to the extent of producing negative Jacobian values. This provides confidence in the results obtained from the registration process.

Additionally, *p*‐value < 0.05 indicated that the observed difference in median Warping between the two systems was statistically significant for the three spheres (Table 5).

In 4 out of 9 cases, ANZAI yielded median Warping values significantly lower than those of GateCT; similarly, in 4 out of 9 cases, GateCT produced significantly lower Warping magnitudes than ANZAI. One comparison case showed equivalent results between the two systems. This indicates a comparable performance between the two systems, with each showing superiority over the other in specific phase and sphere.

In contrast to the work presented by Kauweloa et al.^2^ our results showed that the surface‐ guided GateCT system revealed its consistency in accurately tracking the respiratory signal. Moreover, in accordance with the investigation of Qubala et al.^14^ which highlighted the superior and consistent respiratory tracking capabilities of SimRT (an updated version of GateCT) compared to ANZAI, our study emphasizes the comparable spatial accuracy provided by both systems. This stands in contrast to the conclusions drawn by Heinz et al.^15^ who found that the Sentinel system did not show results with significant improvement over ANZAI.

### 4.3 Response of the GateCT System Versus Weight

Heinz et al.^15^ reported a CT couch baseline drift on a Toshiba CT scanner, which was found to be dependent on the couch load. However, in our study, despite increasing the couch load from 0 to 103 kg, the breathing signal remained stable using 3 mm/s couch speed. Therefore, the GateCT system was verified to detect the breathing pattern clearly and consistently across different loads using 3 mm/s couch speed on Siemens SOMATOM CT Scanner.

## 5 CONCLUSIONS

This study compared two respiratory monitoring systems for 4DCT reconstruction using deformable image registration as an assessment approach. Quantitative metrics such as DSC, MSD, and AVE were comparable for both systems, in accordance with the guidelines provided by AAPM TG132. The consistent estimates of both systems DSC close to 1 across the three spheres of the respiratory phantom, with the MSD < 2 mm, indicated the similarity of spheres contours of the moving phantom with the contours of the static phantom. This agreement showed an accurate spatial and temporal estimation of contour volumes resulting from the two systems. The insignificant results of the *t*‐test for the anatomical metrics suggested that the metrics obtained when using the GateCT and ANZAI systems were comparable across the three spheres. In the realm of deformation field metrics, the ANZAI system, characterized by median Jacobian values close to 1, demonstrated efficacy in mitigating volume deformation during respiration. Furthermore, both systems exhibited comparable performance in terms of median Warping magnitude across the three spheres. Finally, our results demonstrated that the ANZAI system and GateCT were comparable in terms of reliability and accuracy in supplying 4DCT with respiratory information, despite the differences in their tracking method. In clinical practice, the combination of a Siemens CT scanner with either ANZAI or GateCT system will provide comparable results in 4DCT reconstruction. Thus, the outcome of this study suggested a promising approach for GateCT™ as an effective tool to assess respiratory motion for clinical applications and successful adoption of deformable image registration methods in assessing 4DCT reconstruction.

## AUTHOR CONTRIBUTIONS

**Ali Al‐Zein**: Conceptualized the study, designed the methodology, and contributed to the manuscript writing. **Rawan H. Naim**: analyzed the data and contributed to the interpretation of results and manuscript writing. **Wassim Jalbout**: designed the methodology and supervised the research. **Bilal Shahine**: conceptualized the study, designed the methodology, supervised the research, provided critical revisions, and approved the final version of the manuscript.

## CONFLICT OF INTEREST STATEMENT

The authors declare no conflicts of interest
